# Characterizing the Evolution of Inter-Actor Networks in the South China Sea Arbitration via Entropy-Driven Graph Representation Learning from Massive Media Event Data

**DOI:** 10.3390/e28030347

**Published:** 2026-03-19

**Authors:** Menglan Ma, Hong Yu, Peng Fang

**Affiliations:** 1 School of Journalism and Information Communication, Huazhong University of Science and Technology, Wuhan 430074, China; menglanma@hust.edu.cn; 2 School of Computer Science and Technology, Huazhong University of Science and Technology, Wuhan 430074, China

**Keywords:** inter-actor networks, information entropy, GDELT, graph representation learning, South China Sea Arbitration, massive media data

## Abstract

On 12 July 2016, the ruling on the South China Sea Arbitration was announced and rapidly drew worldwide attention, turning the event into a major international hotspot. Quantifying the dynamics of such hotspot events and understanding the evolution of media-based inter-actor networks during major shocks are of substantial research interest. Viewing these interactions as dynamic networks, we analyze the time-varying actor interaction structure surrounding the arbitration using the Global Database of Events, Location and Tone (GDELT), a large-scale media-based event database with global coverage since 1979. We extract nearly 30,000 events related to the arbitration from 5 July to 25 July 2016, constructing daily cooperation and conflict networks to quantify structural changes via network size and degree-entropy dynamics. To further reveal actor-level structural roles, we learn node embeddings on each daily network via an entropy-driven graph representation learning scheme and perform embedding-based clustering with automatically selected cluster numbers, visualized via t-SNE. The results show that key dates in the event window are associated with pronounced structural shifts in the networks, including changes in participation breadth, degree-distribution heterogeneity, and clearer differentiation and reconfiguration of actor roles, with distinct patterns between cooperation and conflict networks. These findings demonstrate the potential of massive media event data for characterizing structural responses and actor-role evolution in event-driven inter-actor networks.

## 1. Introduction

The South China Sea dispute concerns sovereignty and territorial integrity [1]. Driven by political and economic interests and strategic interactions among multiple parties, the dispute has become increasingly internationalized and multilateral [2]. Following the announcement of the South China Sea Arbitration ruling on 12 July 2016, the event rapidly drew worldwide attention, evolved into a prominent international hotspot, and further intensified regional tensions. This gives rise to a key research question: how can we quantitatively characterize the evolution of inter-actor networks before and after the arbitration, especially focusing on how different actors, particularly China, respond during the event? Addressing this question is practically important for foreign policy making [3], including safeguarding sovereignty and territorial integrity while easing tensions and promoting peaceful coexistence and development with neighboring actors.

Media-based actor interactions can be viewed as complex networks [4] whose structures may change substantially when major events occur. This motivates event-centric network evolution analysis [5], where temporal variations in network size, connectivity, and structural heterogeneity are used to describe relational dynamics around a shock. However, for hotspot events involving many interacting actors and mixed cooperative and conflict signals, purely topology-level indicators can be insufficient for interpreting which actors change structural roles, how, and when [6]. To bridge this gap, we integrate information-theoretic characterization [7] with representation learning [8] and analyze actor-level role differentiation and reconfiguration along the event timeline.

A prerequisite for constructing such networks is a comprehensive and reliable data source. The Global Database of Events, Location and Tone (GDELT) (https://www.gdeltproject.org/(accessed on 1 January 2026)) records massive media-based event data worldwide and has been increasingly used in machine learning [9] and computational social science [10], including measuring country activity [11], detecting and forecasting domestic political crises [12], modeling dynamic financial networks [13], and analyzing global disaster coverage [14]. It has also been used to study conflict dynamics and support hotspot identification [15,16]. Meanwhile, recent studies have further summarized the development and application landscape of the GDELT event database and highlighted practical considerations such as data redundancy and key-field accuracy [17], motivating careful preprocessing and robust modeling when using GDELT in event-centric analyses [18]. In this work, we use GDELT to study how media-based inter-actor networks evolve around the South China Sea Arbitration, and we examine both graph topology and learned representations to characterize structural changes across the event timeline.

Network science has established canonical structural properties such as random graphs [19], small-world phenomena [20], and scale-free behavior [21], and has been widely adopted in sociology and other disciplines [22]. Beyond static structure, empirical studies have shown that temporal evolution of networks can reflect event-driven dynamics, for example in earthquake [23] and online social networks [24]. More recently, graph representation learning has provided systematic tools to encode graph entities (e.g., nodes) into low-dimensional vectors that preserve relational and structural information [25], offering a complementary perspective to classic topology-only analysis [26]. In our study, the arbitration period naturally yields a sequence of daily snapshots; accordingly, we learn day-specific node embeddings and leverage their day-to-day variation to interpret actor-level structural roles and their reconfiguration, rather than forecasting beyond the observed window. Throughout the paper, we use the term “countries and regions” to denote network actors.

**Contributions.** This paper makes four contributions. (1) We construct daily cooperation and conflict relation networks among relevant actors from GDELT event data and quantify network evolution using time series of network size and degree-entropy dynamics. (2) We propose an entropy-driven graph representation learning pipeline to learn daily node embeddings that capture latent structural roles; our design is motivated by recent progress on entropy-based graph representation learning [27,28,29,30,31]. (3) We perform embedding-based clustering (with automatically selected cluster numbers) and visualization to reveal actor groupings and role differentiation, and we compare how these patterns differ between cooperation and conflict networks. (4) We empirically show that key event dates are associated with pronounced structural shifts and actor-role reconfiguration, demonstrating that combining entropy-based indicators with representation learning can effectively characterize the structural evolution of media-based inter-actor networks during hotspot events.

The remainder of this paper is organized as follows. Section 2 introduces the South China Sea Arbitration and the GDELT dataset. Section 3 presents the construction of daily inter-actor networks, the degree-entropy measure, and the entropy-driven graph representation learning pipeline. Section 4 reports empirical results on network evolution and embedding-based actor grouping. Section 5 discusses and concludes the paper.

## 2. Materials

### 2.1. The South China Sea Arbitration

The South China Sea Arbitration refers to the arbitration initiated by the Republic of the Philippines, which claimed that China’s maritime entitlements asserted under the “nine-dash line”, together with China’s law-enforcement activities and island and reef development in the disputed waters of the South China Sea (referred to by the Philippines as the West Philippine Sea), violated the United Nations Convention on the Law of the Sea (UNCLOS). The arbitration was conducted under Annex VII of UNCLOS. The arbitral tribunal was composed of arbitrators appointed in accordance with the relevant procedures, and the proceedings were carried out with administrative support provided by the Permanent Court of Arbitration in The Hague.

On 22 January 2013, the then-government of the Philippines unilaterally initiated the arbitration concerning disputes between China and the Philippines in the South China Sea. On 12 July 2016, the tribunal issued its final award on the case. According to the award, the Philippines prevailed. The five arbitrators unanimously held that, under UNCLOS, China does not have “historic rights” to natural resources in the South China Sea based on the “nine-dash line”. The award also addressed environmental impacts associated with land reclamation activities and called for the cessation of certain activities in the South China Sea.

The ruling was announced on 12 July 2016. In addition to this key date, several events around the ruling further shaped the event trajectory. First, on 6 July, China’s Ministry of Foreign Affairs stated at a regular press conference that the military exercises conducted in the South China Sea from 5 July to 11 July were not targeted at any specific country, in response to Vietnam’s accusation on 4 July that China had infringed upon Vietnam’s sovereignty. Second, on 6 July, the Asia–Europe Meeting (ASEM) concluded in Ulaanbaatar, Mongolia, where participating parties agreed that maritime disputes should be resolved in accordance with international law. Third, on 18 July, China’s Maritime Safety Administration announced that the People’s Liberation Army would conduct a three-day military exercise in the South China Sea starting on 19 July. Finally, on 24 July, the 49th ASEAN Foreign Ministers’ Meeting opened in Vientiane, Laos. China’s Foreign Minister Wang Yi attended the China–ASEAN (10 + 1) Foreign Ministers’ Meeting held there, and on 25 July a joint statement on the “Comprehensive and Effective Implementation of the Declaration on the Conduct of Parties in the South China Sea” was released, encouraging the parties to address differences peacefully through dialogue and consultation.

### 2.2. Overview of the GDELT Event Database

The Global Database of Events, Language, and Tone (GDELT) is a terabyte-scale media-based event database that records event information derived from worldwide news coverage. Its sources include hundreds of thousands of broadcast, print, and online news outlets across the globe, with the source list expanding over time. GDELT covers news records from 1 January 1979 to the present, spanning both English and non-English media (over 100 languages), and reports broad geographic and language coverage. In GDELT, each event record is processed into a set of structured attributes. In particular, each record contains two actors and a Goldstein scale score. Here, an actor denotes the participating entity (in our study, an actor corresponds to a country or a region), and the Goldstein scale measures the degree of cooperation or conflict between the two actors: positive values indicate cooperation, whereas negative values indicate conflict [32].

### 2.3. South China Sea Arbitration Event Data

**Figure 1:**
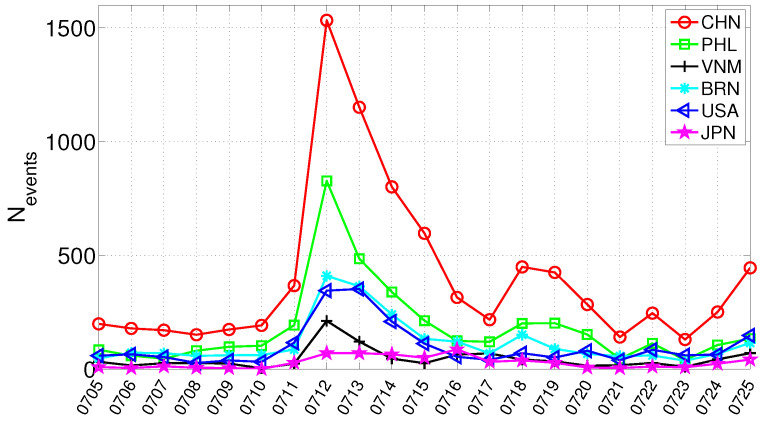
Daily event volume for major actors (countries and regions) in the South China Sea Arbitration.

Based on news sources indexed by GDELT, we extracted nearly 30,000 event records related to the South China Sea Arbitration from 5 July 2016 to 25 July 2016 (a total of 21 days), which defines our event window. The event window was selected to match the temporal evolution of the South China Sea Arbitration. Specifically, it covers the pre-ruling buildup, the ruling announcement on 12 July 2016, and the immediate post-ruling response period. This choice is also consistent with the time series of South China Sea Arbitration-related events in GDELT, where event activity is concentrated around this period. In this study, network nodes are not taken directly from free-text entity mentions in news reports. Instead, they are constructed from standardized actor codes in GDELT, where actors are encoded under the CAMEO framework as structured event participants. We retain actor codes that can be mapped to clear geopolitical actors. Most of these correspond to countries, while a small number correspond to region-level geopolitical actors that appear as independent actor codes in the underlying data coding framework; for this reason, we use the term “countries and regions” in this manuscript. For example, in this study, Taiwan is part of China and is treated as a region-level actor only in our empirical analysis because the underlying data coding represents it as a separate actor code, and retaining this coded actor helps characterize how different actors respond within the event network. General subnational administrative units are not modeled as separate actors. Figure 1 shows the time series of the number of reports associated with major participating actors during this period. The observations indicate that China is one of the primary actors in the arbitration, and the volume of China-related reports is substantially higher than that of other actors. Therefore, excluding China is treated as an analytical design choice rather than a rule of actor coding. This is intended to better capture the response patterns of the broader international community and to mitigate the disproportionate influence of China-related reporting volume on the constructed networks. This design helps reveal the response patterns and structural differentiation among other actors more clearly, but it also means that the resulting networks focus on the broader inter-actor response structure after removing the dominant focal actor. Accordingly, we exclude events in which China appears as an actor from subsequent analyses.

## 3. Methods

### 3.1. Constructing International Relation Networks Among Countries and Regions

Each media-based event record in GDELT contains two actors and a *Goldstein scale,* which quantifies the extent of cooperation (positive values) or conflict (negative values) between the two actors. Based on the extracted South China Sea Arbitration event set, we construct a daily event matrix (i.e., a weighted adjacency matrix) for each day *t*:

(1) $$A\left(t\right)={\left(w_{ij}\left(t\right)\right)}_{n\times n}$$

where *n* denotes the number of actors (countries and regions) involved, and $w_{ij}\left(t\right)$ denotes the aggregated interaction strength between actor *i* and actor *j* on day *t*, obtained by aggregating the Goldstein scores of all events involving the pair on that day.

Since GDELT events include both cooperative and conflict interactions, we construct two weighted matrices per day: a cooperation matrix $A^{\left(P\right)}\left(t\right)$ and a conflict matrix $A^{\left(N\right)}\left(t\right)$. Let $m^{\left(P\right)}$ and $m^{\left(N\right)}$ denote the number of cooperation (Goldstein >0) and conflict (Goldstein <0) events, respectively, between actors *i* and *j* on day *t*. We define the daily cooperation and conflict weights as:

(2) $$w_{ij}^{\left(P\right)}\left(t\right)=\sum_{k=1}^{m^{\left(P\right)}}GS_{k}^{\left(P\right)}\left(t\right),$$

(3) $$w_{ij}^{\left(N\right)}\left(t\right)=\sum_{k=1}^{m^{\left(N\right)}}GS_{k}^{\left(N\right)}\left(t\right),$$

where $GS_{k}^{\left(P\right)}\left(t\right)$ and $GS_{k}^{\left(N\right)}\left(t\right)$ are the Goldstein scores of the *k*-th cooperation and conflict events between *i* and *j* on day *t*, respectively. To mitigate event-volume heterogeneity, these summed weights are further normalized by the corresponding number of events between actors *i* and *j* on day *t*, and the resulting average weights are used in subsequent analyses. This normalization helps reduce the inflation caused by highly uneven event counts, but it does not fully remove media coverage bias. Accordingly, the resulting weights should still be interpreted as media-reported interaction signals.

Major hotspot events may induce substantial temporal variations in relation structures. Therefore, we construct a sequence of daily cooperation and conflict matrices from 5 July to 25 July 2016. We adopt day-level networks to match the native granularity of GDELT events and to facilitate event-centric comparisons along the arbitration timeline. The resulting daily snapshots provide an analytical slicing of a temporally continuous relational process; temporal dependence such as persistence, delayed responses, and reporting carry-over may exist across adjacent days. Accordingly, our analysis focuses on descriptive characterization of structural variation around key milestones and window-based comparisons, rather than fine-grained attribution to a single calendar day.

In each daily network, nodes represent actors (countries and regions), and the network is constructed as an undirected weighted graph. An edge (i,j) exists if there is at least one event between *i* and *j* on day *t*, and its weight is given by the corresponding entry in the relevant daily matrix (i.e., $w_{ij}\left(t\right)$, or $w_{ij}^{\left(P\right)}\left(t\right)$ and $w_{ij}^{\left(N\right)}\left(t\right)$ for cooperation and conflict, respectively). In this study, such edges do not represent formal state positions or international relations in a stronger substantive sense; instead, they encode actor-pair cooperation/conflict interaction strengths aggregated from GDELT media events within the selected time window, and therefore reflect media-reported interaction structures. The degree of node *i* is defined as the number of its nonzero connections, i.e., the number of nonzero entries in the *i*-th row (or column) of the corresponding daily matrix.

### 3.2. Degree Entropy of Daily Networks

In a network, nodes and edges jointly determine its structure. Let $d_{i}\left(t\right)$ denote the degree of node *i* on day *t*, and let *n* be the number of nodes. We define the degree-based probability of node *i* as

(4) $$p_{i}\left(t\right)=\frac{d_{i}\left(t\right)}{D_{\mathrm{total}}\left(t\right)},\;D_{\mathrm{total}}\left(t\right)=\sum_{i=1}^{n}d_{i}\left(t\right)$$

Then, the information entropy of the degree distribution for the daily network is

(5) $$H\left(t\right)=-\sum_{i=1}^{n}p_{i}\left(t\right)\log p_{i}\left(t\right)$$

where log denotes the natural logarithm (base *e*). Intuitively, H(t) reflects the heterogeneity of the daily network in terms of the degree distribution, i.e., how evenly node degrees are distributed. Accordingly, we use degree entropy as a macro-level descriptor to summarize how connectivity is allocated across actors in each daily snapshot. Our macro-objective is to compare network states across days and event windows in terms of whether interactions become more evenly distributed or more concentrated on a subset of actors. The degree distribution provides a first-order and directly interpretable summary of such connectivity allocation, and entropy offers a parsimonious, comparable indicator of its heterogeneity.

### 3.3. Entropy-Driven Graph Representation Learning

To move from macro-level structural characterization to micro-level role analysis, we adopt a framework that combines macro-level network descriptors with graph representation learning. This design allows us to characterize both structural shifts in inter-actor networks and the role similarity and differentiation of actors across daily snapshots. To complement topology-level descriptors (e.g., network size and degree entropy), we further learn node embeddings for each daily snapshot. Graph representation learning maps nodes to low-dimensional vectors, so that relational and structural patterns in the original graph can be analyzed in a continuous space. Such embeddings are widely used in downstream tasks including recommendation [33], link prediction [34], and node classification [35].

**Figure 2:**
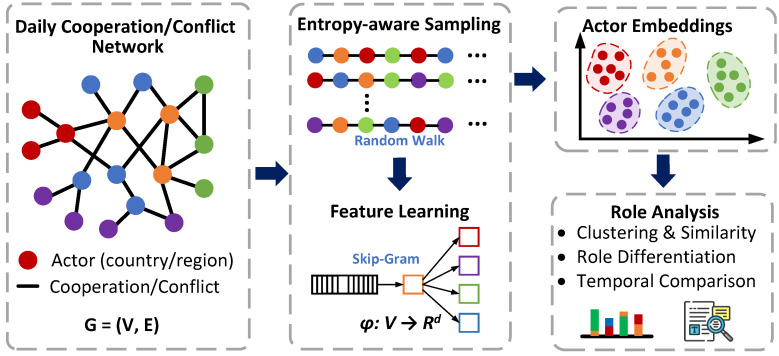
Conceptual overview of the entropy-driven embedding pipeline.

**Overview of the entropy-driven embedding pipeline.** Figure 2 provides a conceptual overview of the embedding workflow used in this study. Starting from each daily cooperation/conflict network, we first generate node sequences through entropy-aware random walks that favor structurally informative co-occurrences. These sampled sequences are then used to train node embeddings with a Skip-Gram objective. The resulting low-dimensional vectors encode actor similarity in the network and are further used for clustering, similarity analysis, and temporal comparison across daily snapshots. In this way, the embedding pipeline serves as a micro-level complement to the macro-level network descriptors, allowing us to examine how actor roles are grouped, separated, and reorganized over time. We adopt an entropy-oriented embedding scheme inspired by HuGE [28]. Compared with classic random-walk-based methods (e.g., node2vec [36] and DeepWalk [37]), HuGE emphasizes *information efficiency* during sampling: it adjusts the walk transition and the sampling budget using entropy-related criteria, aiming to reduce redundant contexts while retaining structurally informative co-occurrences. Following [28], the embedding pipeline consists of two stages: (i) sampling node sequences from the graph via entropy-aware random walks; and (ii) learning node vectors from the sampled sequences using a Skip-Gram objective.

**Entropy-aware random walk sampling.** This sampling design is intended to preserve two kinds of structural cues that are relevant to our empirical task: similarity of interaction neighborhoods and differences in actor prominence within the daily network. Given the current node *u*, a candidate next-hop node *v* is drawn from the neighbor set N(u). HuGE first assigns an unnormalized transition score that jointly reflects (a) local structural similarity via common neighbors and (b) the influence of high-degree nodes:

(6) $$\alpha \left(u,v\right)=\frac{1}{deg(u)-Cm(u,v)}\times \max\left\{\frac{deg(u)}{deg(v)},\frac{deg(v)}{deg(u)}\right\}$$

where deg(·) denotes node degree and Cm(u,v) is the number of common neighbors between *u* and *v*. Intuitively, $\frac{1}{deg(u)-Cm(u,v)}$ increases with more common neighbors (with fixed deg(u)), and the degree ratio term highlights transitions involving high-degree nodes [38,39].

To avoid scanning all neighbors in each step, HuGE uses a *walking-backtracking* acceptance rule [39]. Specifically, the candidate *v* is accepted as the next-hop node with probability

$$P\left(u,v\right)=Z\left(\alpha \left(u,v\right)\right),\;Z\left(x\right)=\frac{e^{x}-e^{-x}}{e^{x}+e^{-x}}$$

If *v* is rejected (with probability 1−P(u,v)), the walker backtracks to *u* and repeats the sampling from N(u) until a candidate is accepted. For weighted graphs, the acceptance probability is extended to

P(u,v)=Z(α(u,v)·w(u,v))

**Entropy-guided walk length.** To avoid overly long and redundant walks, HuGE adaptively stops sampling once additional steps contribute limited new structural information. Instead of using a fixed walk length for all nodes, HuGE terminates a walk when additional steps contribute little new information. Consider a walk from source node *u* with length *L*:

$W_{u}^{L}=\left\{v_{u}^{1},v_{u}^{2},\ldots ,v_{u}^{L}\right\}$. Let n(v) be the number of occurrences of node *v* in $W_{u}^{L}$. The entropy of the walk is

(7) $$H\left(W_{u}^{L}\right)=-\sum_{v\in W_{u}^{L}}\frac{n(v)}{L}\log\frac{n(v)}{L}$$

As *L* increases, $H(W_{u}^{L})$ typically grows and then stabilizes once the visitation distribution becomes steady. HuGE quantifies the (diminishing) linear association between $H(W_{u}^{L})$ and *L* using the coefficient of determination ($R^{2}$) and stops the walk when the correlation becomes sufficiently small:

(8) $$R\;\left(H(W_{u}^{L}),L\right)=\frac{\sum_{i=1}^{L^{*}}\left(H\left(W_{u}^{L(i)}\right)-\bar{H(W_{u}^{L})}\right)\left(L\left(i\right)-\bar{L}\right)}{\sqrt{\sum_{i=1}^{L^{*}}{\left(H\left(W_{u}^{L(i)}\right)-\bar{H(W_{u}^{L})}\right)}^{2}}\sqrt{\sum_{i=1}^{L^{*}}{\left(L\left(i\right)-\bar{L}\right)}^{2}}}$$

where $\bar{H(W_{u}^{L})}$ and $\bar{L}$ denote the means of the respective series for $1\leq i\leq L^{*}$, and $L^{*}$ is the resulting walk length. In practice, HuGE uses $R^{2}\left(H,L\right)<\mu$ as the termination condition and sets μ=0.995 [28].

**Entropy-guided number of walks.** Let $p\left(v\right)=\frac{deg(v)}{\sum_{v\in V}deg\left(v\right)}$ denote the degree distribution, and let $q\left(v\right)=\frac{ocn(v)}{\sum_{v\in V}ocn\left(v\right)}$ denote the empirical occurrence distribution of nodes in the sampled corpus, where ocn(v) is the number of times node *v* appears in the corpus. HuGE measures the mismatch between *q* and *p* via relative entropy and increases the number of walks per node until the improvement becomes negligible. Specifically, as the number of walks *r* increases, the change converges:

(9) $$\Delta D_{r}(q∥p)=\left|D_{r}(q∥p)-D_{r-1}(q∥p)\right|$$

and HuGE stops increasing *r* once $\Delta D_{r}(q∥p)\leq \delta$, where δ=0.001 in [28].

**Feature learning.** Given the sampled walks, embeddings are learned by optimizing a Skip-Gram objective, as in node2vec [40]. The learned vectors provide low-dimensional actor representations that are subsequently used to analyze role similarity, clustering structure, and temporal role differentiation. Let $\varphi :V\to R^{d}$ map each node to a *d*-dimensional vector. Using a context window size *w*, the objective maximizes the co-occurrence likelihood of nodes appearing within the window along the walks:

(10) $$\underset{\varphi }{\mathrm{argmax}}\;\frac{1}{\left|V\right|}\sum_{j=1}^{\left|V\right|}\sum_{-w\leq i\leq w}\log p\left(u_{j+i}|u_{j}\right),$$

where $u_{j+i}$ is a context node of $u_{j}$ and $p(u_{j+i}|u_{j})$ is typically parameterized by a softmax function. In our implementation, we set the embedding dimension to d=128 and the context window size to w=10, following the parameter setting used in HuGE [28].

## 4. Results

### 4.1. Event Trajectory Characterization via Goldstein Scale

**Figure 3:**
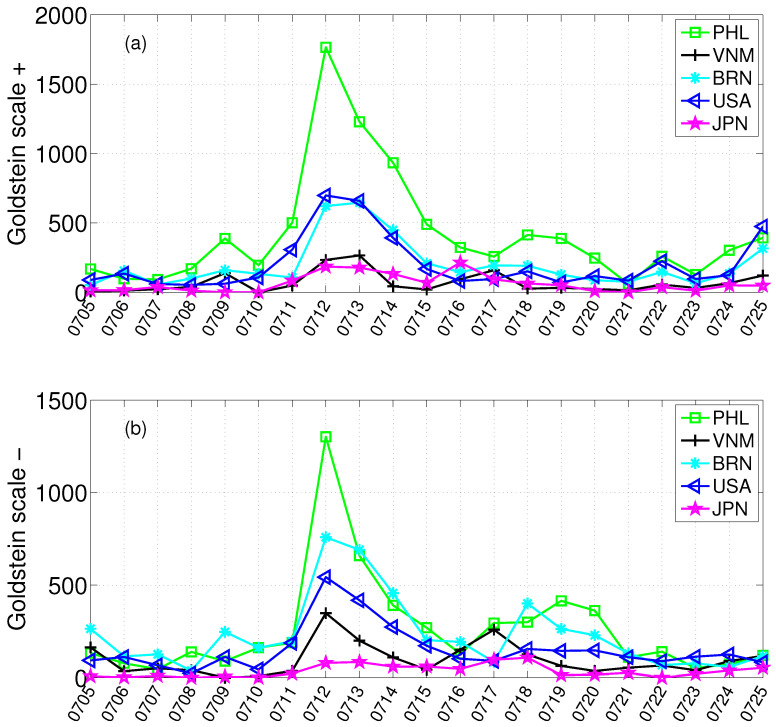
Daily Goldstein-scale time series of cooperative (**a**) and conflictive (**b**) events for major actors in the South China Sea Arbitration.

The final ruling on the South China Sea Arbitration was announced on 12 July 2016. To obtain an initial quantitative view of cooperation and conflict signals during the event window, we aggregate the Goldstein scale over all arbitration-related events on each day. Figure 3 reports the resulting daily time series from 5 July to 25 July. A pronounced surge appears on 12 July, when both the cooperation and conflict series increase sharply and reach their highest levels within the observation window. This suggests that the ruling announcement immediately intensified media-reported interactions in both positive and negative directions. After 12 July, the two series decline from the peak but continue to fluctuate, indicating that the event remained highly salient in the following days rather than ending as a one-day shock. Several secondary rises can still be observed in the post-ruling period, suggesting that subsequent diplomatic statements and related developments continued to reshape the media-event dynamics. Overall, the Goldstein-scale time series provides a useful macro-level characterization of the event trajectory and helps identify key dates of intensified interaction. At the same time, these aggregate signals alone cannot reveal how inter-actor networks are reorganized, which actors become structurally prominent, or how role differentiation evolves over time. We therefore next turn to network-based structural analysis.

### 4.2. Evolution Analysis of Inter-Actor Networks

#### 4.2.1. Macro-Level Structural Shifts in Cooperation and Conflict Networks

**Figure 4:**
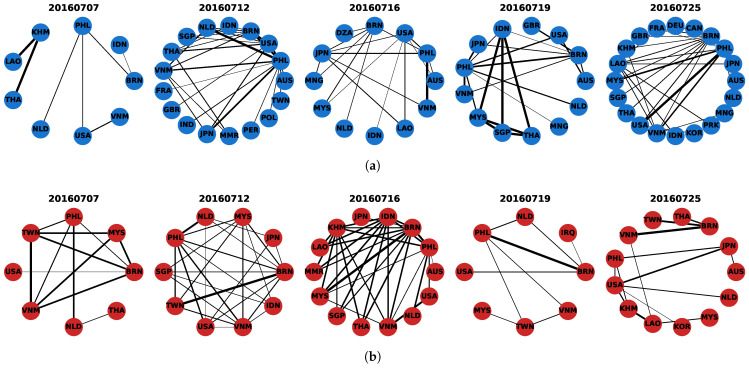
Representative daily cooperation and conflict networks in the South China Sea Arbitration on key dates (7, 12, 16, 19, and 25 July 2016). Nodes denote countries and regions, and edges denote media-event-based cooperation or conflict interaction signals between actor pairs. (**a**) Cooperation networks; (**b**) Conflict networks.

**Figure 5:**
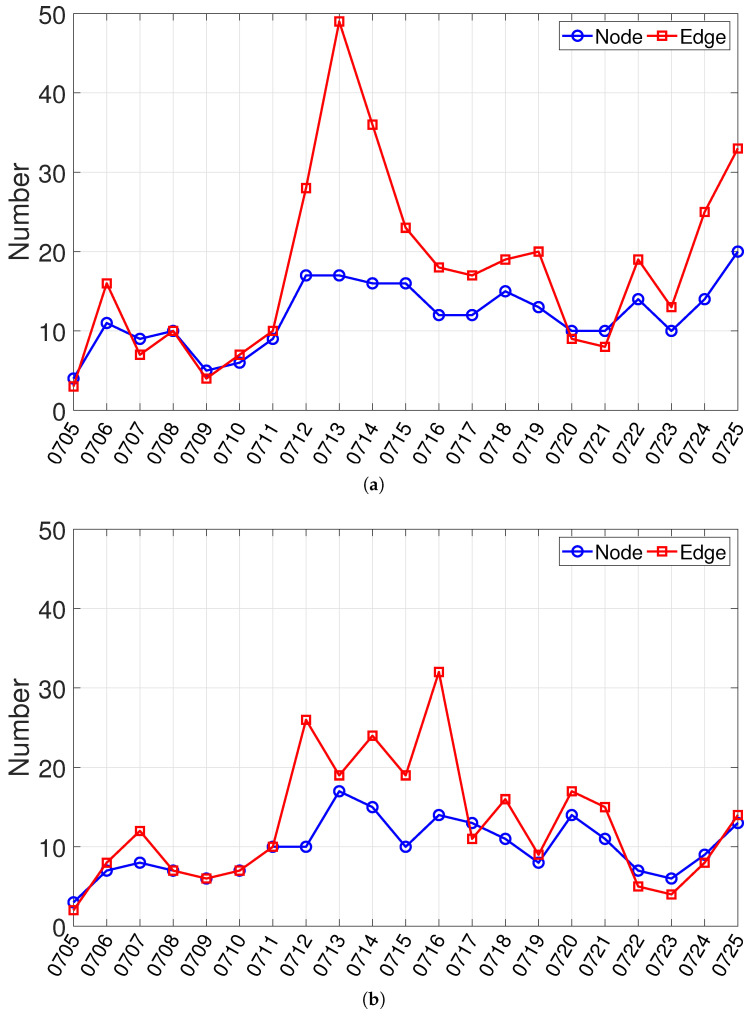
Time series of daily cooperation (**a**) and conflict (**b**) networks in the South China Sea Arbitration. The blue line with circle markers denotes the number of nodes, and the red line with square markers denotes the number of edges.

To characterize the short-term evolution of inter-actor networks around the South China Sea Arbitration, we first examine macro-level structural changes in the daily cooperation and conflict networks. Figure 4 visualizes the revised networks on five representative key dates (7, 12, 16, 19, and 25 July 2016). In these networks, nodes denote countries or regions, and edges denote media-event-based actor-pair cooperation or conflict interaction signals observed on a given day. The representative snapshots already suggest noticeable variation in both the scope of participation and the density of interactions across the event window. To quantify these changes, we use two topology-level indicators: the number of nodes and the number of edges in each daily network. Here, the node count reflects the breadth of actor participation, while the edge count reflects the density of observed interaction signals. Figure 5 reports the corresponding time series for the cooperation and conflict networks and reveals several dates on which the network structure changes markedly. Both networks exhibit clear structural shifts around several key dates, especially 12 July, 16 July, and 25 July. 12 July marks the most prominent joint escalation around the ruling announcement, with both networks showing clear expansion relative to the preceding days. In the cooperation network, the number of edges further rises to its maximum on 13 July (49 edges), while the number of nodes reaches its highest level on 25 July (20 nodes), indicating that the post-ruling stage not only intensified cooperation signals but also broadened the range of participating actors. In the conflict network, the edge count reaches its maximum on 16 July (32 edges), suggesting that conflict-coded interactions became particularly dense in the immediate post-ruling period. By contrast, the network on 19 July shows a more selective and locally concentrated structure, indicating that the interaction pattern had shifted from broad expansion to more focused structural reorganization. The networks in Figure 4 are broadly consistent with these temporal patterns. The networks on 12 July are visibly denser than those on earlier dates, while the conflict network on July 16 shows a particularly concentrated and highly connected structure. By 25 July, the cooperation network expands again, consistent with the increase in participating actors observed in Figure 5. Overall, these size-based indicators provide a first macro-level screening of structural shifts over the event window. They help identify the dates on which participation breadth and interaction density change most visibly.

#### 4.2.2. Quantifying Network Dynamics with Degree Entropy

**Figure 6:**
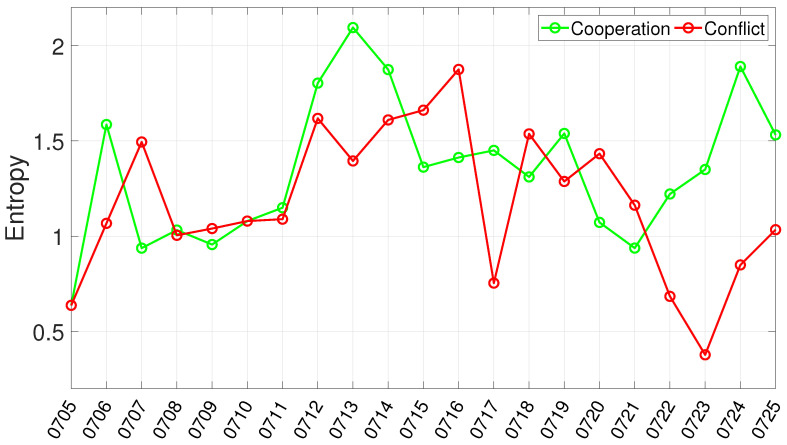
Time series of degree entropy for the daily cooperation and conflict networks in the South China Sea Arbitration.

Network size captures the breadth of participation and the density of observed interactions, but it does not reveal how these interactions are distributed across actors. To address this, we compute the degree entropy of each daily network, which reflects the heterogeneity of degree allocation. In our setting, degree entropy serves as a macro-level indicator of degree-distribution heterogeneity, characterizing whether connectivity is more evenly distributed across actors or more concentrated on a subset of them over time. Figure 6 shows the entropy time series for the cooperation and conflict networks. Several prominent fluctuations can be observed over the event window. In the cooperation network, entropy rises sharply around 12–14 July and again around 24 July, indicating that cooperation-related connectivity becomes more broadly distributed across actors during these periods. In the conflict network, entropy remains relatively high around 12–16 July and then exhibits another increase around 18–20 July, suggesting that conflict-coded interaction patterns were also redistributed across a wider set of actors in the immediate post-ruling stage. By contrast, lower entropy values indicate periods in which interaction links are concentrated on a smaller number of actors, implying a more uneven connectivity structure. Compared with the node and edge counts in Figure 5, the entropy series adds a different layer of structural information. While size-based indicators identify when participation breadth and interaction density change most visibly, degree entropy reveals whether these changes are broadly shared across actors or concentrated on a limited subset of them. We therefore use this macro-level trend to contextualize the embedding-based micro-analysis.

### 4.3. Micro-Level Analysis of Inter-Actor Role Differentiation

**Figure 7:**
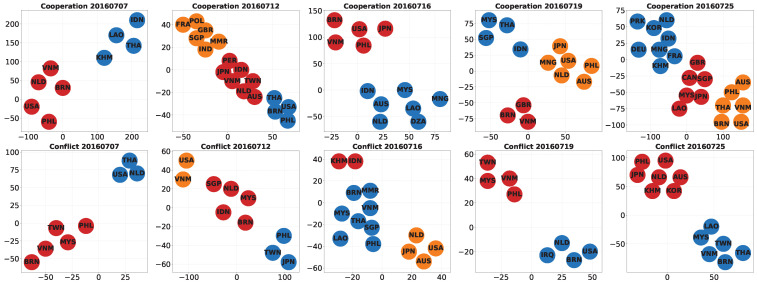
Node embeddings and clustering results based on entropy-driven graph representation learning, comparing cooperation and conflict networks on representative key dates. Nodes represent countries and regions, and colors indicate cluster memberships.

To complement the macro-level descriptors (node counts, edge counts, and degree entropy) and to further examine which actors exhibit similar interaction patterns across key periods, we apply entropy-driven graph representation learning to derive node embeddings for each daily cooperation and conflict network. We then perform K-means clustering [41] on the embedding space, with the number of clusters selected automatically using the silhouette score. Specifically, we search *k* from 2 to 10, perform K-means clustering for each candidate *k*, and choose the value that maximizes the average silhouette coefficient. Next, we use t-SNE [42] to project the embeddings into a two-dimensional space to visualize the structural similarity between actors, as shown in Figure 7. The 2D coordinates are used only for visualization, whereas the relative distance and cluster membership in the high-dimensional embedding space reflect similarities in interaction partners and interaction intensity patterns. Therefore, the embedding space provides a micro-level view of actor roles and their differentiation across key dates.

**(1) Differentiation around the ruling.** Before the ruling, the cooperation network exhibits relatively coarse clustering in the embedding space. On 7 July, the actors are grouped into a limited number of broad clusters, suggesting that cooperation-related interaction patterns were still relatively concentrated and had not yet developed into a highly differentiated structure. By contrast, on 12 July, the day of the ruling announcement, the cooperation embedding space becomes visibly more segmented, with multiple distinct clusters and clearer separation among actor groups. This pattern suggests that the ruling announcement coincided not only with an increase in interaction volume, as shown in the macro-level results, but also with an expansion in the range of structural roles played by different actors in the cooperation network. In substantive terms, this indicates that the event did not simply intensify attention, but also generated more differentiated cooperation-related responses across actors, including clearer grouping tendencies among actors exposed to similar diplomatic or agenda-related signals. A similar but even sharper pattern appears in the conflict network. On 12 July, the conflict embeddings display stronger inter-cluster separation and a more fragmented arrangement than on 7 July, indicating that conflict-coded interaction patterns rapidly differentiated after the ruling. Rather than interpreting this as a direct measure of formal political alignment, we view it as evidence that actors were being positioned into more distinct media-reported conflict interaction roles during the immediate post-ruling stage. The sharper separation also suggests that conflict-related responses were becoming less diffuse and more clearly segmented across different groups of actors.

**(2) Reconfiguration in the post-ruling stage.** If 12 July marks a phase of rapid differentiation, 16 July and 19 July are more indicative of structural reconfiguration. In the cooperation network, the positions of several actors shift noticeably between these two dates, and the embedding space becomes increasingly reorganized into multiple groups, especially on 19 July. This suggests that the cooperation network did not simply stabilize after the initial shock of the ruling, but instead continued to reorganize in association with subsequent diplomatic developments and security-related narratives in the event stream. This pattern is more consistent with agenda reorganization than with a simple return to the pre-ruling state: actors appear to regroup as the focus of interaction moves from the ruling itself to follow-up diplomatic and security-related concerns. The conflict network shows a related but distinct pattern. Around 16 July and 19 July, cluster separation remains pronounced, but the relative positions of several actors change across the embedding space. This indicates that conflict-coded interaction roles were not fixed after the ruling announcement but continued to be reassembled as the media-event context evolved. In this sense, the embedding results reveal not only differentiation but also the dynamic reshaping of actor roles across the post-ruling period. Such reshaping suggests that conflict narratives remained contested and that the boundaries between different response groups were still being actively reconfigured.

**(3) Divergent evolution of cooperation and conflict.** By 25 July, when the joint statement by China and ASEAN foreign ministers was released, the cooperation network showed tighter grouping among several actors in the embedding space. Compared with the more dispersed configuration on 19 July, this pattern suggests a certain degree of regrouping or convergence in cooperation-related interaction roles. In more substantive terms, this may reflect a temporary convergence of cooperation-related agendas, with some actors becoming more closely aligned in the media-reported interaction space. At the same time, the conflict network retains a clearly segmented cluster structure, indicating that conflict-coded interaction roles remain differentiated rather than collapsing back into a simple structure. This suggests that while cooperation-related responses show signs of regrouping, conflict-related responses remain segmented and less easily reconciled. Taken together, these patterns do not indicate a fully convergent response across the actor network. Instead, they suggest a limited convergence in cooperation-related interactions, while conflict-related differences remained present in the media-reported interaction space. From this perspective, the post-ruling evolution is better understood as a phase of temporary stabilization than as a clear return to a unified response structure. This distinction further enriches the earlier macro-level findings by showing that cooperation- and conflict-related interactions did not evolve in parallel, even when they were associated with the same major event.

**(4) Macro–Micro correspondence.** The embedding results also help explain the macro-level entropy patterns reported in Figure 6. Periods with higher-degree entropy, such as around 12–16 July and again near 24–25 July in the cooperation network, tend to coincide with broader dispersion and clearer differentiation in the embedding space. Likewise, in the conflict network, periods of elevated entropy are accompanied by stronger separation among actor groups. This correspondence suggests that degree entropy captures a macro-level shift in how connectivity is distributed across actors, while the embedding analysis reveals how that shift unfolds in terms of actor-role differentiation and reorganization. Overall, the micro-level embedding analysis complements the earlier macro-level results by showing that the key dates identified through nodes, edges, and degree entropy are associated not only with changes in participation breadth or interaction density, but also with shifts in actor-role differentiation and reorganization. Together, these results form a coherent macro–micro evidence chain for understanding structural responses in the South China Sea Arbitration.

## 5. Discussion and Conclusions

The final ruling on the South China Sea Arbitration, announced on 12 July 2016, rapidly attracted global attention, leading to a sharp increase in the event’s significance and sparking widespread controversy, further escalating tensions in the South China Sea region. Media-based inter-actor interactions can be viewed as a dynamic network of relations among multiple countries and regions, and major international events often coincide with noticeable disturbances and reconfiguration in these network structures. Based on this understanding, this study extracts nearly 30,000 event records related to the South China Sea Arbitration from the GDELT media database, covering the period from 5 July to 25 July 2016. It constructs daily cooperation and conflict networks and provides a quantitative characterization of their structural evolution over the event window.

The study reveals the significant potential of using massive media event data for quantifying international hotspot events. First, the time series of the Goldstein scale for cooperation and conflict events effectively captures the fluctuations in international attention over time and highlights several key time points. However, given the large number of countries or regions involved in the South China Sea Arbitration, and the highly complex interactions among them, relying solely on Goldstein-scale data is insufficient to further reveal the evolution of inter-actor interaction patterns and the mechanisms of role differentiation. In contrast, by constructing daily inter-actor networks and analyzing their structural evolution, we can more effectively track the development process of the event and uncover how media-reported interaction structures change around major milestones.

At the network level, we construct daily cooperation and conflict networks, quantify network size changes by counting the number of nodes and edges, and use degree entropy to measure degree-distribution heterogeneity. The results indicate that key time points identified by these structural indicators align closely with the actual event timeline, corresponding to events such as China’s Foreign Ministry’s response to Vietnam’s claims on 6 July, the announcement of the ruling on 12 July, the Asia–Europe Meeting (ASEM) on 16 July, the military exercises held by China’s navy in the South China Sea from 19 July to 21 July, and the joint statement released by China and ASEAN foreign ministers on 25 July. Overall, the time series of network size and degree entropy provides a useful macro-level characterization of the evolving inter-actor networks related to the South China Sea Arbitration and captures several phase shifts in the event trajectory.

Furthermore, to overcome the limitation of macrostructural indicators, which cannot answer questions like “which actors form similar interaction structural roles around key time points, and how do these roles evolve and reconfigure over time”, this study introduces an entropy-driven graph representation learning method to learn daily node embeddings. K-means clustering is then applied to the embeddings, with automatic tuning for selecting the optimal number of clusters. The results show that the number of clusters and the degree of separation between actors in the embedding space increase noticeably around key event dates, reflecting a transition from relatively coarse structural grouping to a more differentiated multi-role configuration. Particularly after the ruling on 12 July, both cooperation and conflict networks have exhibited significant refinement in structural roles and group differentiation, suggesting that the ruling announcement coincided not only with heightened interaction intensity but also with short-term reorganization in actor interaction roles. Moreover, the cooperation and conflict networks show distinct clustering patterns around key time points, demonstrating a dual-track pattern of structural evolution: cooperation exhibits tendencies of agenda convergence and structural cohesion, while conflict interactions tend to show enhanced inter-cluster separation and persistent structural polarization. Taken together, the graph learning-based micro-analysis provides a more interpretable actor-level evidence chain that complements the earlier macro-level results from the network size and degree entropy.

In summary, this study proposes a framework for analyzing the evolution of media-based inter-actor networks around major international events by constructing daily cooperation and conflict networks, identifying key time points with macro-level indicators such as network size and degree entropy, and further combining graph representation learning with clustering analysis to reveal the differentiation and reconfiguration of actor structural roles. This framework is particularly suitable for event-driven, time-sensitive, media-based inter-actor network analysis, where the goal is to understand structural responses and actor-role reorganization around major geopolitical events.

This study has several limitations. First, GDELT is a media-based event dataset and may therefore reflect reporting bias, coding inconsistency, and incomplete contextual information. Although the event-count normalization used in this study helps mitigate part of the bias associated with uneven event volumes, it cannot fully eliminate media coverage effects. As a result, the constructed networks should be interpreted as media-reported interaction structures rather than the full underlying reality of inter-actor relations. Second, both international interactions and media reporting may exhibit temporal dependence, including persistence of ties, delayed responses, and reporting carry-over across adjacent days. Accordingly, the day-level networks in this study are best understood as an analytical slicing for event-centered, window-based structural comparison. We do not adopt a more explicit temporal model because the present study focuses on event-centered structural comparison and actor-role interpretation around key milestones, rather than temporal prediction or causal time series modeling. Explicit temporal dependency modeling would be a valuable extension for future work, especially for studying temporal smoothing or forecasting. Accordingly, the observed network changes are interpreted as patterns associated with major milestones rather than strict day-specific causal effects.

## Data Availability

The data supporting the results of this study are publicly available from the GDELT database (https://www.gdeltproject.org/ (accessed on 1 January 2026)) and were used under the terms of the GDELT data access policy.
